# Sex Differences in Long-term Outcome of Prenatal Exposure to Excess Glucocorticoids—Implications for Development of Psychiatric Disorders

**DOI:** 10.1007/s12035-023-03522-5

**Published:** 2023-08-10

**Authors:** Frederik Elberling, Stefan Spulber, Raj Bose, Hoi Yee Keung, Virpi Ahola, Zongli Zheng, Sandra Ceccatelli

**Affiliations:** 1 Department of Neuroscience, Karolinska Institutet, Biomedicum, Solnavägen 9, 171 77 Stockholm, Sweden; 2 Ming Wai Lau Centre for Reparative Medicine, Karolinska Institutet, 15W Science and Technology W Ave, Sha Tin, Hong Kong Special Administrative Region, People’s Republic of China

**Keywords:** Depression, Sex differences, ADHD, Circadian, Suprachiasmatic nucleus

## Abstract

Exposure to prenatal insults, such as excess glucocorticoids (GC), may lead to pathological outcomes, including neuropsychiatric disorders. The aim of the present study was to investigate the long-term effects of in utero exposure to the synthetic GC analog dexamethasone (Dex) in adult female offspring. We monitored spontaneous activity in the home cage under a constant 12 h/12 h light/dark cycle, as well as the changes following a 6-h advance of dark onset (phase shift). For comparison, we re-analysed data previously recorded in males. Dex-exposed females were spontaneously more active, and the activity onset re-entrained slower than in controls. In contrast, Dex-exposed males were less active, and the activity onset re-entrained faster than in controls. Following the phase shift, control females displayed a transient reorganisation of behaviour in light and virtually no change in dark, while Dex-exposed females showed limited variations from baseline in both light and dark, suggesting weaker photic entrainment. Next, we ran bulk RNA-sequencing in the suprachiasmatic nucleus (SCN) of Dex and control females. SPIA pathway analysis of ~ 2300 differentially expressed genes identified significantly downregulated dopamine signalling, and upregulated glutamate and GABA signalling. We selected a set of candidate genes matching the behaviour alterations and found consistent differential regulation for ~ 73% of tested genes in SCN and hippocampus tissue samples. Taken together, our data highlight sex differences in the outcome of prenatal exposure to excess GC in adult mice: in contrast to depression-like behaviour in males, the phenotype in females, defined by behaviour and differential gene expression, is consistent with ADHD models.

**Supplementary Information:**

The online version contains supplementary material available at 10.1007/s12035-023-03522-5.

## Introduction

Prenatal insults have been associated with an increased risk of psychiatric disorders [1]. Exposure to excess glucocorticoids (GC), due to maternal stress, placenta enzymatic failure or treatment with synthetic GC, is among the events posing a risk for depression in adult life [2], but the mechanisms behind the induced detrimental effects are not completely understood. While there is accumulating evidence on sex-related differences in susceptibility to various neurodevelopmental toxic stimuli [3–5], there is very limited knowledge on sex-related differences in GC-induced effects. The importance of gender-related differences is highlighted by brain RNA-sequencing studies revealing sex differences at the transcriptional level in several psychiatric disorders [6, 7], including depression [8] and ADHD [9]. Biological differences between sexes emerge as research progresses in scope and depth, yet investigations considering both sexes are still lagging [10].

Psychiatric disorders have been associated with altered circadian rhythms and sleep–wake cycle [11, 12]. External interference with circadian rhythms, such as sleep deprivation, shift work, light deprivation, or constant artificial light, is associated with an increased risk of depression in humans as well as in animal models [13–15]. The synchronisation of behaviour and metabolism with the 24-h light–dark cycle is achieved via the central clock, located in the suprachiasmatic nucleus (SCN) in the anterior hypothalamus [16]. The core mechanism consists of interlocked transcription and translation feedback loops which generate self-sustained oscillations with a period of approximately 24 h [17]. While almost all cell types in the mammalian organism express a functional molecular clock mechanism, synchronisation across cells requires constant entrainment by the SCN, and rhythmic secretion of GC plays a critical role [18]. In rodents reared in laboratory environment, the main circadian entrainer is environmental light (photic entrainment). Photic entrainment requires direct input to the SCN from the retina via the retinohypothalamic tract (RHT) and dopamine signalling from the ventral tegmental area (VTA) [19, 20]. The SCN acts on the hypothalamic–pituitary–adrenal (HPA) axis by release of arginine vasopressin (AVP) from axon terminals in the hypothalamic paraventricular nucleus (PVN), and the subsequent rhythmic secretion of GC from the adrenal gland further entrain peripheral oscillators [21, 22]. Accumulating evidence indicates that there are sex-related differences in circadian rhythms and their regulation [23–27].

We have investigated the effects of in utero exposure to the synthetic GC analog dexamethasone (Dex) in a mouse model, where pregnant mice are injected with Dex during the last week of gestation. This treatment induces intrauterine growth retardation (IUGR) [28, 29], a condition associated with neuropsychiatric disorders, such as depression [30] and attention-deficit hyperactive disorder (ADHD) [31, 32]. Male offspring exposed to Dex in utero displayed alterations in photic entrainment of spontaneous activity which preceded the onset of depression-like behaviour [33]. They also present rigid patterns of activity under normal LD cycle, and the onset of activity was re-entrained immediately after advancing the onset of dark by 6 h. Furthermore, there was a lower amplitude of oscillations in GC secretion and downregulated GC receptor (GR), resulting in a weaker coupling of peripheral tissue with the SCN. Dex-exposed mice did not respond to antidepressant treatment with Fluoxetine (SSRI class antidepressant) but did respond to desipramine (DMI; SNRI class antidepressant), which also restored the coupling between SCN and peripheral oscillators by enhancing GR signalling [34]. Moreover, DMI treatment at 6 months of age prevented the onset of depression-like behaviour at the age of 12 months [35]. It has been shown that transient exposure to Dex in mid-gestation induces sex-specific alterations in placental gene expression regulation [36], suggesting potential sex-related differences in impact on developmental processes. In light of this background, we directed our investigations towards the effects of prenatal exposure to Dex in female mice. We first analysed the impact of advancing the onset of dark phase on home cage behaviour; then, we explored gene expression regulation in the SCN and the potential connections with alterations in behaviour. Taken together, our study reveals that adult female mice exposed to Dex in utero exhibit behavioural traits observed in ADHD mouse models.

## Materials and Methods

### Animals and Treatment

Experiments were conducted in agreement with the European legislation and Swedish national regulation following approval by the local Animal Ethics Committee (Stockholms Norra djurförsöksetiska nämnd, ethical permit N1/15).

Dexamethasone (Dex) was diluted in sterile saline solution to a final concentration of 5 µg/ml. Pregnant C57Bl/6NCrl dams (*N* = 5 females/group) (CharlesRiver, SCANBUR Research, Sollentuna, Sweden) were injected subcutaneously with either 50 µg/kg/day Dex or an equivalent volume of vehicle (10 ml/kg/day) from gestational day 14 up until delivery (PND0). This dose induces a mild decrease in bodyweight until about 3 weeks after birth in both male and female pups [28, 33]. On post-natal day 3 (PND3), all litters were culled to 4 pups per litter to include both males and females, if possible. The mice were weaned at PND21, and the animals were tagged subcutaneously with a radio frequency identification transponder (RFID) (Trovan™ Unique T-100A, Trovan, UK) under brief 4% isoflurane anaesthesia. RFID tags allowed unambiguous identification of individual animals during the experiments in the home cage. At weaning, the animals were placed into groups of up to 5 animals per cage, with each animal originating from a different litter. Male and female pups underwent the same procedures, and the reported number of animals for each experiments represents the number of litters.

### Forced Swim Test

We evaluated depression-like behaviour in both male and female mice aged 12 months (*N* = 8 per group) by means of forced swim test (FST), as described elsewhere [33–35]. Briefly, the animals were individually placed for 6 min in plastic cylinders (24 cm height, 12 cm diameter) filled with water (23.5 °C) to a depth of 16 cm. After testing, the mice were dried with paper towels and returned to the home cage to recover under additional heating provided by an infrared lamp. The experiments were videotaped, and the footage was analysed offline by one investigator who was blind to the treatment and exposure conditions. Immobility was defined as continuous passive floating lasting for at least 2 s. The total immobility time over the last 5 min of recording was used for subsequent analyses.

### Recording of Spontaneous Behaviour

Spontaneous activity of mice (*N* = 5/group) was recorded with the TraffiCage system (NewBehavior, Zurich, Switzerland) as described previously [33, 35]. The TraffiCage system is comprised of an array of five antennas placed underneath the cage. The antennas read the RFID tag from each mouse providing a location with a resolution of 20 ms. For activity monitoring, the animals were placed in GR900 cages (Tecniplast, Buguggiate, Italy) inside a sound- and light-isolating, climate-controlled enclosure (Scantainer, Scanbur, Sollentuna, Sweden) (22 ± 1 °C; 50 ± 5% relative humidity), with a 12:12 h light/dark (LD) cycle (light intensity 200 lx) and food and water ad libitum. The interaction with human researchers was limited to cage changes (which include refreshing water and food) once per week. Each recording session included two cages monitored simultaneously, one with control and one with Dex-exposed animals. The cages were placed randomly on either of two TraffiCage plates inside the enclosure, and the position and orientation of each cage were maintained throughout the experiment. The animals were allowed 1 week to acclimatise to the enclosure after the move from the housing facility, and to ensure full entrainment to the LD cycle in the enclosure. Baseline behaviour was recorded for a minimum of 3 days before the introduction of a 6-h phase advance (advance onset of dark phase), and the recording continued during the phase shift and the following 5 days.

### Analysis of Spontaneous Activity

The availability of continuous monitoring of spontaneous activity in the home cage environment provides the opportunity to follow the activity in both active and inactive phases, as well as across consecutive LD cycles. In addition, monitoring the activity of several animals provides insight into the social aspects of spontaneous behaviour.

#### Entrainment of Spontaneous Activity

The time series of activity counts were exported as ASCII files and analysed using custom algorithm implementations in Matlab™ (Version 9.3 and above; The MathWorks™, Natick, MD, USA) (see also [33, 35]). To identify the onset of the active phase, the activity was binned in 5 min non-overlapping epochs and smoothed with a sliding Gauss window (2 h width). The epochs with activity above the individual’s detrended average were considered “active epochs”. The active phase was defined as a sequence of active epochs contiguous or separated by gaps no larger than 1 h. The onset of the active phase was defined as the time corresponding to the beginning of the active phase (relative to subjective time, ZT; ZT0 = light on). The activity recording was smoothed with a sliding Gauss window spanning 243 consecutive bins (20.25 h), and the circadian peak of activity was defined as daily maxima. We also evaluated the distribution of activity between light and dark phases by calculating the proportion of total visits occurring during the dark phase (dark bias). The calculation of dark bias yields the following special values: 0 – all activity recorded in the light phase; 0.5 – activity equally distributed in light and dark; 1 – all activity recorded in the dark phase. The synchronisation of activity in group-housed animals was estimated as the uncorrected coefficient of determination (Pearson *R*^2^) for linear regression of each animal’s time series of activity against the time series of activity of the other animals housed in the same cage within 24-h intervals.

Further analyses based on the classification of visits and location within the cage were performed using a custom algorithm implemented in RStudio (version 4.0.5; RStudio, PBC, Boston, MA, USA). Recordings were first resampled at 1 s resolution to align timelines across individual animals. To provide a consistent reference point for analysis of movement inside the home cage, we first identified intervals when all mice were detected by the same antenna and defined the antenna with the longest time spent together as the nest. The four other antennas were then annotated accordingly to their position in relation to the “nest” antenna as “adjacent”, “across”, “diagonal” and “middle”. This was done for each LD cycle to capture potential relocations of the nest during the observation time. A “visit” was defined as the time interval spent by an individual animal in the detection area of a single antenna. To differentiate active visits from resting visits, we applied a threshold. According to Blumberg et al. [37], a threshold can be defined based on the distribution of visits. When visits are plotted semi-logarithmic duration vs. frequency, inactive bouts would exhibit a linear distribution, whereas active visits would have an exponential distribution. The linear inactive part could then be determined through linear regression (*R*^2^ > 0.96 and the standard deviation of slope < 0.05), enabling us to find the threshold between active and inactive visits (~ 7 min overall average) (Supplementary Fig. S1A). A sequence of visits below the threshold was then classified as “trip”, punctuated by “rest” intervals (above the threshold) (Supplementary Fig. S1B).

We applied summary statistics (count, average, standard deviation and skewness) on active (trips) and inactive (rest) behaviours for each light and dark phase independently. We considered the time the mouse spent alone, with one other animal or with more than one animal. The trips were also classified based on the location of start and end antennas into four subtypes: trips starting and ending in the nest antenna (NN), trips starting in the nest antenna and ending in one of the other four antennas (NR), starting outside of the nest and ending in the nest (RN) and finally trips starting and ending outside of the nest (RR). Rest intervals were classified based on occurrence in the nest area or elsewhere in the cage (i.e. any other antenna than the nest). This procedure generated 129 variables (see Supplementary Table 1) for each animal for each light and dark section each day. During the light section of the phase shift day, counts were multiplied by 2 to minimize the impact on downstream analysis since this day only yielded 6 h of behaviour during subjective day (light phase). Each variable was then tested within each group to eliminate features with no variance across groups. For downstream application, we defined the LD cycle of the advance in the onset of the dark phase as d0; we included 3 to 5 days before d0 to evaluate baseline behaviour, and 5 days (d + 1 to d + 5) after d0 to evaluate re-entrainment. The data was log-normalized [log10(1 + variable/10)] before further calculations.

#### Affinity Propagation Clustering

Affinity propagation (AP) clustering is an unsupervised machine-learning algorithm based on the concept of “passing messages between datapoints” which has the advantage of unbiased determination of both number of clusters and cluster composition [38]. The purpose of using AP clustering was to identify consistent similarities between variables across groups, without explicitly aiming to assign biological interpretations to individual clusters. First, we applied AP clustering (package in RStudio) on scaled data consisting of baseline (individual features’ average before the phase shift) and 6 days following the phase shift. The values for each feature were pooled across treatment, sex and light/dark, yielding 129 sets with 266 entries each (19 animals × 7 days/animal × 2 phases/day). The sample quantile (*q*) was set to 0.23 as indicated by the optimization steps searching for a range where the number of clusters was stable. This procedure yielded 18 clusters consistent across groups (Supplementary Fig. S2A). The group belonging was then used for FDR correction when analysing the difference between Dex and control mice in the following steps: analysis of organisation of behaviour at baseline, and changes thereof following the phase shift. To analyse the effects of prenatal exposure to Dex on the organisation of behaviour at baseline, we calculated the *z*-score relative to control for males and females independently. To analyse the effects of phase shift on individual features, we calculated the *z*-score difference from baseline for each animal and day and used FDR-corrected paired, two-sided *t* test (Supplementary Fig. S2B).

#### Uniform Manifold Approximation and Projection

Uniform manifold approximation and projection (UMAP) is a non-linear dimensionality reduction algorithm which allows 2D visualisation of multidimensional data, in which distances between individual points are meaningful in relation to the actual distances in the features’ hyperspace [39]. Briefly, the resulting maps allow the quantification of differences between behaviour patterns considering all 129 variables. In RStudio, we applied the “umap” package after scaling the data to create UMAP coordinates using the following settings: nearest neighbours = 25; min dist = 0.3; number of components = 2; number of epochs = 500; and spread = 1. These values were selected after extensive testing across different parameter ranges to yield sufficient separation between light and dark behaviour on the UMAP coordinate map. The coordinates were used for downstream analysis of individual trajectories as follows: sequences of individual projections in the UMAP plane were analysed separately for light and dark. For each sequence, we computed a distance matrix between all points. We then estimated distances from baseline by averaging all distances between each point and baseline (Supplementary Fig. S3A, B).

### Sample Preparation for RNA-Sequencing and qPCR

Mice were sacrificed with an overdose of anaesthetic (sodium pentobarbital 150 mg/kg, i.p.) and perfused transcardially with ice-cold phosphate-buffered saline. All animals were sacrificed within 1 h after the offset of subjective night. To avoid light exposure, the animals were transported from the holding facility in light-proofed cages about 3 min before killing. The brains were removed and snap frozen on dry ice, then stored at –80 °C. For the collection of SCN and hippocampal samples, the brains were placed in a cryostat (− 25 °C) for 30 min before the dissection to reheat. Samples from the dorsal hippocampus were obtained using a dissection needle (diameter 1 mm, depth 1 mm) after removing the cortex with a scalpel blade. The SCN was dissected by needle puncture (diameter 1 mm; depth 1 mm) immediately caudal of the optic chiasm. Dissected tissue was placed for 20 min in hypotonic buffer N (10 mM Hepes pH 7.5, 2 mM MgCl_2_, 25 mM KCl), and after placed in the Biomasher III (Nippi, Japan) for tissue homogenisation. The flowthrough was collected after spinning down and mixed gently in a sucrose solution (2 M). The solution was spun down (4 °C) by 6000 RFC for 10 min. The supernatant was removed, and nuclei were resuspended in ice-cold Buffer N (hypotonic buffer N, with 2 M sucrose, 1 mM DTT and 1 mM PMSF) and spun down again for 10 min. Finally, the supernatant was removed, and the nuclei were resuspended in a freezing solution (3:7 buffer N in glycerol) and stored at − 80 °C for further analysis.

#### Bulk RNA-Sequencing

RNA from isolated brain regions was extracted using Monarch RNA Miniprep kit (NEB, England), according to the manufacturer’s recommendations. RNA concentration of each sample was measured using Qubit RNA Assay Kit with Qubit 2.0 Fluorometer (Life Technologies, USA). Then, cDNA sequencing libraries were constructed using Collibri 3′ mRNA Library Prep Kit for Illumina (Invitrogen, USA) following the manufacturer’s guidelines. Briefly, mRNA was reverse transcribed with oligodT primer containing an Illumina-compatible sequence at its 5′ end. After the first strand synthesis, RNA was removed by heat treatment, and the second strand synthesis was initiated with random primers containing Illumina-compatible linker sequence at its 5′ end. Finally, the libraries were amplified with Illumina’s adapters for cluster generation and indices for multiplexing. Constructed libraries were quantified by qPCR using the Collibri Library Quantification kit (Invitrogen, USA) on StepOnePlus Real-Time PCR System (Applied Biosystems, USA). Paired-end reads of 150-bp long were generated on NovaSeq 6000 platform (Illumina, USA) at Novogene Bioinformatics Technology Co., Ltd., in Beijing, China.

To pre-process the sequenced reads, Illumina TruSeq adapters and poly-A sequences were trimmed from the raw reads and NextSeq-specific quality trimming was applied with Cutadapt [40]. Read quality was evaluated with fastQC [41] before and after trimming. Trimmed reads were mapped to mouse genome (version GRC39) with STAR [42]. Finally, mapped reads were assigned to annotated features and read counts were calculated for each genomic feature using featureCounts function[43] in Rsubread Bioconductor package in R (version 4.1.0). Next, we removed the genes labelled as clock-controlled genes (CCG) (http://cgdb.biocuckoo.org/download.php) to avoid false positive discoveries due to the desynchronisation of the SCN across groups. On the remaining genes, we identified differentially expressed genes (DEGs) between control and Dex-exposed female mice using the DESeq2 package and Wald test in RStudio. After correcting for multiple testing for FDR, we set a cut-off of adjusted *p* value < 0.05 and |log2foldchange|> 1.5 as significantly changed DEGs. The differences in gene expression were also investigated with the signalling pathway impact analysis (SPIA) protocol, which identified 6 significantly altered pathways.

#### Selection of Genes for Expression Analysis

We imported data from the International Mouse Phenotype Consortium (IMPC) to investigate the association between behaviour from known knockout models, matching with not only behaviour in our animals but also the DEGs found in our dataset. These matching genes were further selected only if found in significantly changed SPIA pathways and used for downstream qPCR. We also matched found DEGs with known ADHD genes from the website http://adhd.psych.ac.cn/LiteratureGene.do. From this list, selected genes (Cnr1, Comt, Gsk3b, Th) known to be associated with ADHD were used to investigate further candidate genes that could be included in the analysis. Adding to this, three genes (Narfl, Haghl and Stub1) from 10.1186/s12920-019–0593-5 were also included in qPCR as these genes have been uniquely associated with ADHD in females and could be relevant in our experimental model. Lastly, we included genes that we identified as differentially methylated in neural stem cell cultures exposed to Dex [44]

#### Gene Expression Analysis by qPCR

Total RNA was extracted from SCN and hippocampal samples using RNeasy Mini Kit (QIAGEN, CatNo 74106) following the manufacturer’s instructions. RNA purity was verified by the ratio of absorbance at 260 nm and 230 nm using a NanoDrop 1000 spectrophotometer (ThermoFisher). cDNA was synthesized using a High-Capacity cDNA Reverse Transcription kit (AppliedBiosystems, CatNo. 4268814) starting from 60 ng RNA template. Amplification reactions were performed with 1 µl cDNA, SYBR Green Master Mix (Applied Biosystems, Stockholm, Sweden) and 0.2 µM of forward and reverse primers. The reaction volume was adjusted to 12.5 µl with DEPC water. Negative control samples contained water instead of cDNA. qPCR was performed using a QuantStudio QS5 system (Applied Biosystems). The PCR cycle conditions were 50 °C for 2 min, 95 °C for 10 min, 95 °C for 15 s and 60 °C for 1 min (40 cycles). To evaluate the amplification of a specific sample, final melting curve (from 60 to 95 °C) was added under continuous fluorescence measurements. All expression values were normalized against the housekeeping gene hypoxanthine phosphoribosyltransferase (Hprt) (∆CT = CT_target gene_ – CT_Hprt_). Relative expression levels were calculated as ∆∆CT = ∆CT_Dex_ – ∆CT_Sal_, and relative expression changes were calculated as 2^−∆∆CT^. All experiments were conducted in triplicates and repeated at least three times. qPCR primer sequences are available in Supplementary Table 2.

### Software Accessibility

The software code used can be made available upon request.

### Statistical Analyses

Statistical analyses were performed in RStudio (Data Science Workbench, version 14) or Statistica (TIBCO Software Inc. (2020). R packages and their specific applications are listed in Supplementary Table 3.

## Results

### Sex-Dependent Effects of Prenatal Dex Exposure on Circadian Re-entrainment of Activity

**Fig. 1:**
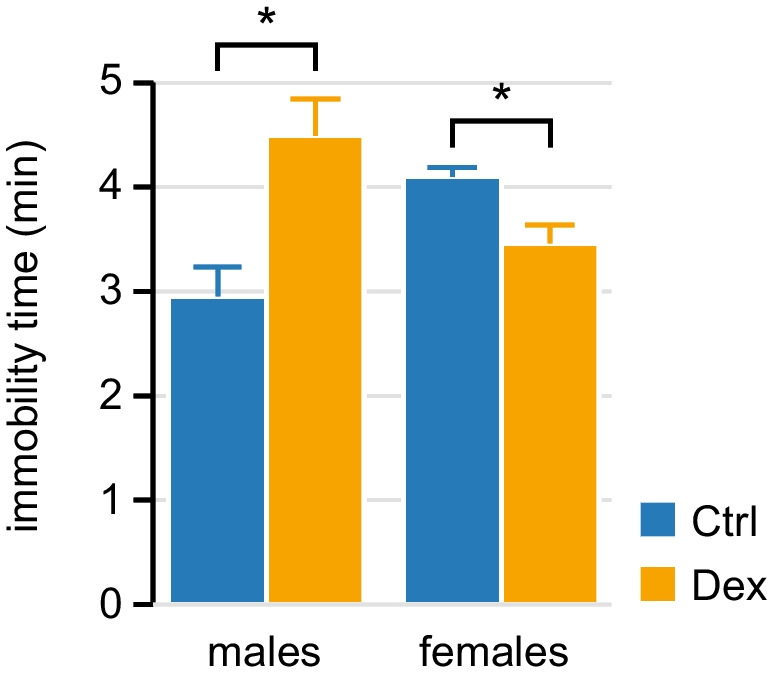
Immobility time in forced swimming test at the age of 12 months. Sex-dependent effects of prenatal exposure to Dex: females exposed to Dex do not display an increase in immobility time indicative of depression-like behaviour, as seen in males

We have reported earlier that prenatal exposure to Dex induces late-onset depression-like behaviour in male mice [33], as well as alterations in circadian entrainment of spontaneous activity before the onset of depression [33, 35]. We tested 12-month-old females in FST and found that, in contrast to males, Dex-exposed females displayed shorter immobility time than controls (Fig. 1). Next, we set out to explore the effects of prenatal exposure to Dex on circadian entrainment of activity in 6-month-old females. We included in analyses the home cage activity data recorded in earlier experiments focusing on male mice [35] in order to highlight the sex-related differences.

**Fig. 2:**
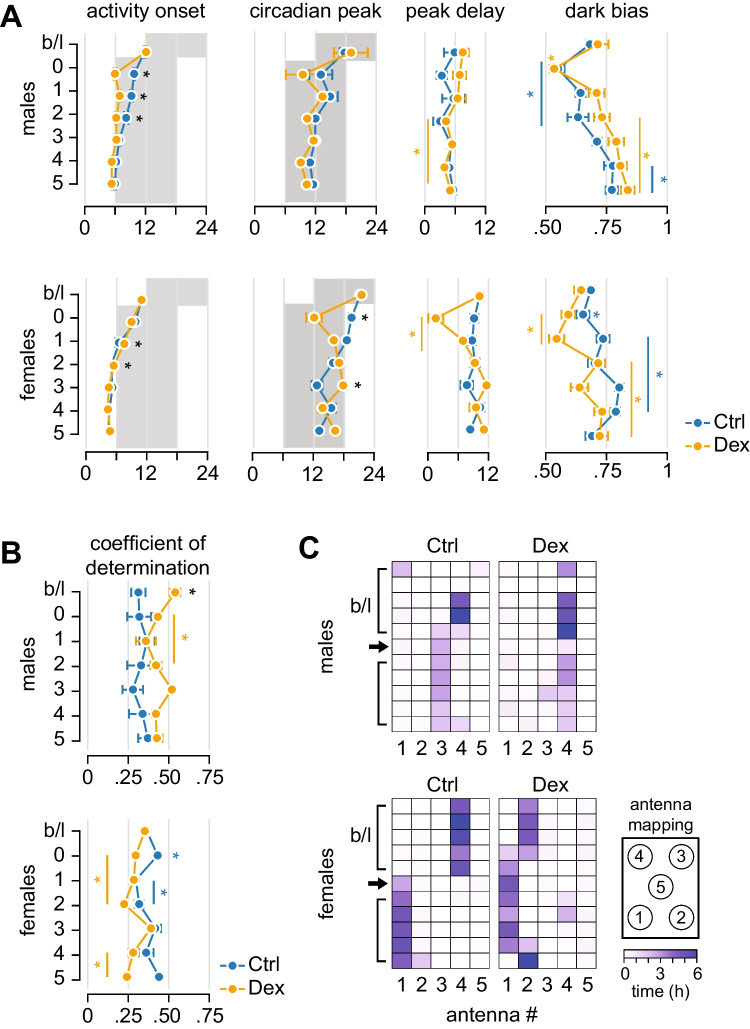
Activity in the home cage after advancing the onset of dark phase by 6 h. **A** Differential re-entrainment of onset and circadian peak of activity. Note the sex-related differences in the effects of prenatal exposure to Dex on circadian re-entrainment: in males, activity onset re-entrains faster in Dex-exposed mice than in controls, and circadian peak of activity follows closely; in females, activity onset re-entrains slower in Dex-exposed mice than in controls, while the circadian peak of activity displays biphasic fluctuations in Dex-, but not in control mice. **B** Synchronization of activity among group-housed mice. In control mice, advancing the onset of dark phase has transient effects only in females, while in Dex-exposed animals (both males and females), the effects persist for several consecutive days. **C** Location of nest antenna as identified by the amount of time the animals spend in a compact cluster. The nest location changes after advancing the onset of dark phase only in controls, while Dex-exposed animals maintain nest location throughout the observation period. Asterisk indicates significant differences; black symbols, difference between groups (repeated measures ANOVA, followed by *t* test); coloured symbols, within-group differences (repeated measures ANOVA followed by Dunnett’s test *vs*. baseline)

First, we investigated circadian re-entrainment of spontaneous activity in response to a 6-h advance in dark phase onset (Fig. 2A) and followed the re-entrainment of active phase onset and circadian peak of activity separately. Dex-males displayed faster re-entrainment of active phase onset, while the circadian peak of activity re-entrained at a similar rate as in controls. In contrast to males, the re-entrainment of active phase onset was slower in Dex-females than in controls, whereas the location of the circadian peak of activity oscillated widely during re-entrainment. The differences in re-entrainment in males and females were emphasized by the variations in delay between activity onset and circadian peak (Fig. 2A). Further support was provided by the variations in the distribution of activity across light and dark phases, assessed as dark bias. In males, Dex-exposed mice exhibited only a brief decrease in dark bias, while in controls, the dark bias was restored to baseline levels after 3 days. Notably, the dark bias increased beyond the baseline in all animals towards the end of the observation period. This is probably a transient effect consistent with more robust entrainment following the phase shift, as described earlier [33].

Next, we asked whether the synchronisation of activity within the cage was affected by phase-advancing the onset of dark. Social interactions can synchronise activity in group-housed animals, provided that the SCN function is preserved [45, 46]. In Dex-exposed males, the synchronisation of spontaneous activity dropped for 3 days following the phase shift, while controls did not display significant differences from baseline (Fig. 2B). Similarly, the synchronisation decreased in Dex-exposed females after the phase shift, while in control animals significantly increased in the first LD cycle following the phase shift, and decreased the following 2 days (Fig. 2B). The changes in synchronisation within the cage suggested that the dynamic adaptation following the phase shift had an impact on social group features. Therefore, we investigated the time that mice spent in specific regions of the home cage, and found that the phase advance of dark onset was followed by a relocation of the nest area (to the adjacent area in males, and to the opposite area in females) in controls, but not in Dex-exposed animals (Fig. 2C).

### Sex-Dependent Effects of Prenatal Dex Exposure on the Organisation of Behaviour

**Fig. 3:**
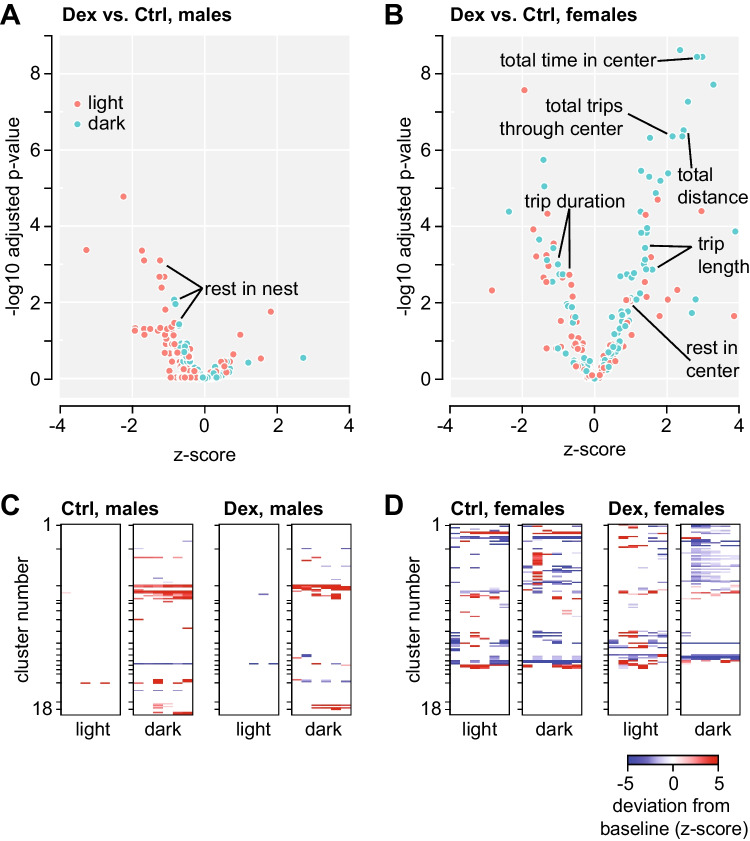
Changes in features (summary statistics of activity in the home cage) after advancing the onset of dark phase by 6 h. **A**, **B** Volcano plots illustrating the magnitude of differences between Dex-exposed mice and controls at baseline, calculated as *z*-score deviations from overall mean for each feature in males (**A**) and females (**B**), respectively. **C**, **D** Longitudinal changes within clusters. Heatmaps showing only significant deviations from baseline. Note that females (**D**) display more widespread differences, both persistent and transient, as compared to males (**C**). See also Supplementary Fig. S2 for details

To evaluate the organisation of behaviour, we extracted 129 features from the recording of spontaneous activity (matching sets of features were extracted for each light and dark phase separately). At baseline, Dex-males displayed overall lower levels of activity (Supplementary Fig. S2), and sparse differences from controls including fewer resting bouts and total duration of rest in the nest area in both subjective day and night (Fig. 3A). In contrast, Dex-females showed overall higher levels of activity than controls (Supplementary Fig. S2), covered longer distance in dark, had longer distance per trip but shorter trip duration, and presented increased activity in the centre of the cage and higher number of inactive visits in light (Fig. 3B). Taken together, this pattern of changes indicates unstimulated hyperactivity and fragmented rest intervals in home cage environment in Dex-exposed females as compared to controls.

To identify features with similar patterns of variation, we used AP clustering (see Supplementary Fig. S3A), then used cluster belonging to apply FDR correction for downstream analyses, without assigning ethological interpretation to individual clusters. The organisation of behaviour following the phase shift showed a larger effect during the dark phase in both sexes, with more widespread changes in females than in males (Fig. 3C, D; see also Supplementary Fig. S3B for details). In addition, the patterns of changes in males appeared to be persistent (i.e. did not return to baseline; Fig. 3C), whereas in females, most features returned to baseline by the end of the observation period (particularly noticeable in clusters #1 and #2; Fig. 3D). Male mice (both controls and Dex) displayed a significant increase in most features in clusters #3 and #17 in dark, suggesting slower spontaneous exploration (increase in visit duration). In females, the highest number of significant alterations in dark phase occurred during the first day after phase shift (d1) and appeared to persist for longer in Dex-females as compared to controls. The transient changes in clusters #1 and #2 indicate a decrease in number of trips in dark and an increase in number of trips in light during re-entrainment in Dex-females, while control females exhibited a brief increase in activity in dark, and otherwise sparse persistent changes in both light and dark. In addition, the persistent decrease in cluster #11 and increase in cluster #12 in dark observed in Dex-females indicate a decrease in time spent in the nest and an increase in rest time in the adjacent area after phase shift. In control females, the persistent decrease in cluster #12 and increase in clusters #13 and #14 points to less rest time in the area adjacent to the nest, but more rest time in the centre of the cage and in areas opposite from the nest area.

**Fig. 4:**
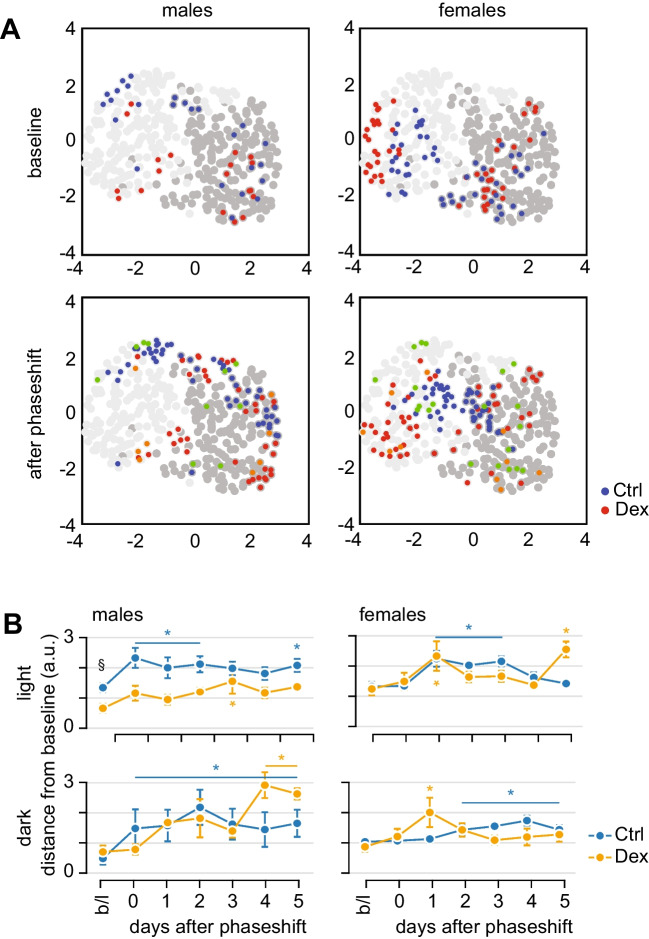
UMAP mapping of behaviour. **A** Mapping of behaviour during light (light grey) and dark (dark grey) phases of the light/dark cycle. Note the overall separation between light and dark behaviour. The separation between Dex-exposed mice and controls at baseline (top panels) is better defined for behaviour in light for both males and females. Following the phase shift (bottom panels), the separation between Dex-exposed males and controls is enhanced in light but reduced in dark. In females, the separation between Dex-exposed and controls is preserved in light, enhanced in dark, but the separation between light and dark is dramatically reduced in controls. Green and orange dots indicate the day of phase shift (d0) for Ctrl and Dex, respectively. **B** Quantification of changes in behaviour following the phase shift as mean Euclidean distance form baseline. Note that females display mostly transient variations, while males display more persistent changes. Asterisk indicates significant differences; black symbols, between-group differences (repeated measures ANOVA, followed by *t* test); coloured symbols, within-group differences (repeated measures ANOVA followed by Dunnett’s test vs. baseline)

Next, we applied uniform manifold approximation and projection (UMAP) to the behavioural features for dimensional reduction to evaluate the effects of advancing the dark on the following light and dark phases. The 2D map did not generate distinct clusters, but we observed a clear separation of light and dark behaviour across both sex and exposure (Fig. 4A). Several features displayed gradients across the separation between patterns of behaviour during subjective day and subjective night (Supplementary Fig S4). The UMAP 2D mapping allows the estimation of distances between individual data points as Euclidean distances in the UMAP plane, provided all data points map within the same cluster. We calculated a matrix of distances between each point and the baseline for light and dark behaviours for each animal (Supplementary Fig. S5A, B). In males, the separation between groups at baseline was clear during the subjective day, but not during subjective night. After phase shift, control males relocated to a different region within the UMAP projections map, in both light and dark, while Dex-males appeared to only relocate during the subjective night. In females, a similar separation of baseline behaviour was observed in both groups, especially during the day. After phase shift, control females’ behavioural changes in both light and dark suggest a reduction in differences between the light and dark phase, i.e. the distance between light and dark behaviour decreases in control females as compared to baseline (Supplementary Fig. S5C). Dex-females showed minor relocation in both light and dark, which was not marked by a similar reduction in the distance between light and dark behaviour (Fig. 4A; Supplementary Fig. S5C). In males, controls displayed persistently increased distance from baseline starting at phase shift day (d0) in both light and dark phases (Fig. 4B). In contrast, Dex-males exhibited virtually no change from baseline in the light phase, and a delayed (starting on d1) persistent deviation in the dark phase. This is consistent with the qualitative observation of differential relocation in the UMAP plane. In females, we found significant differences from baseline starting earliest 1 day after phase shift (d1), consistent with the findings from AP clustering analysis. Dex-females deviated from baseline only on d1 for both light and dark phases, while controls diverged significantly from baseline for 3 consecutive days only in light.

### Differential Gene Expression and Correlations with Behavioural Phenotype in Females

**Fig. 5:**
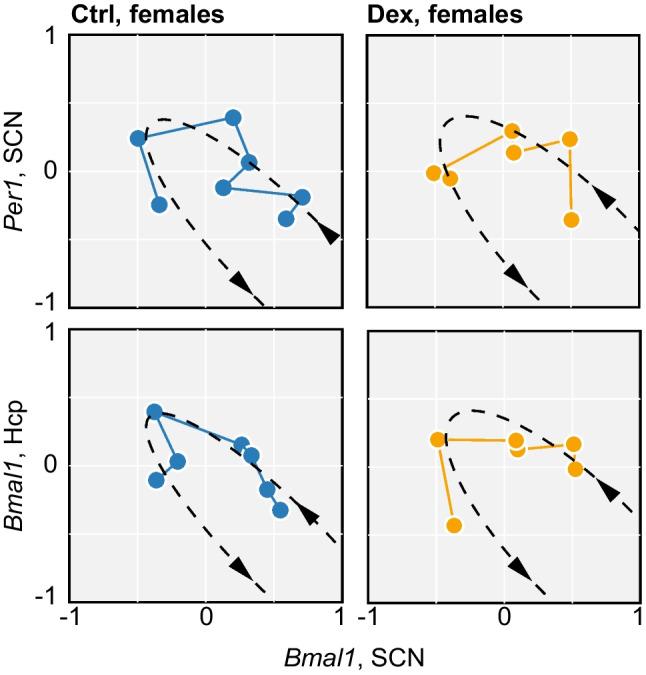
Photic entrainment of SCN and downstream coupling under constant entrainment conditions (at baseline). The relative expression levels for *Bmal1* and *Per1* in the SCN indicate that the molecular clock in the SCN is entrained to the LD cycle in Dex-exposed and in control females alike. Similarly, the coupling between SCN and peripheral oscillators (hippocampus) appears preserved in Dex-females

Photic circadian entrainment of spontaneous activity is driven by the central clock, located in the SCN in the anterior hypothalamus, and requires coupling between SCN and peripheral oscillators [47, 48]. In Dex-males, we found no evidence of altered photic entrainment of the SCN in constant light–dark cycle, but the downstream coupling between hippocampal formation and SCN was impaired [35]. In female mice, we evaluated the expression of core clock genes in the SCN and hippocampus and found no evidence of alterations in either photic entrainment of the SCN, or in downstream coupling between SCN and peripheral oscillators (Fig. 5).

**Fig. 6:**
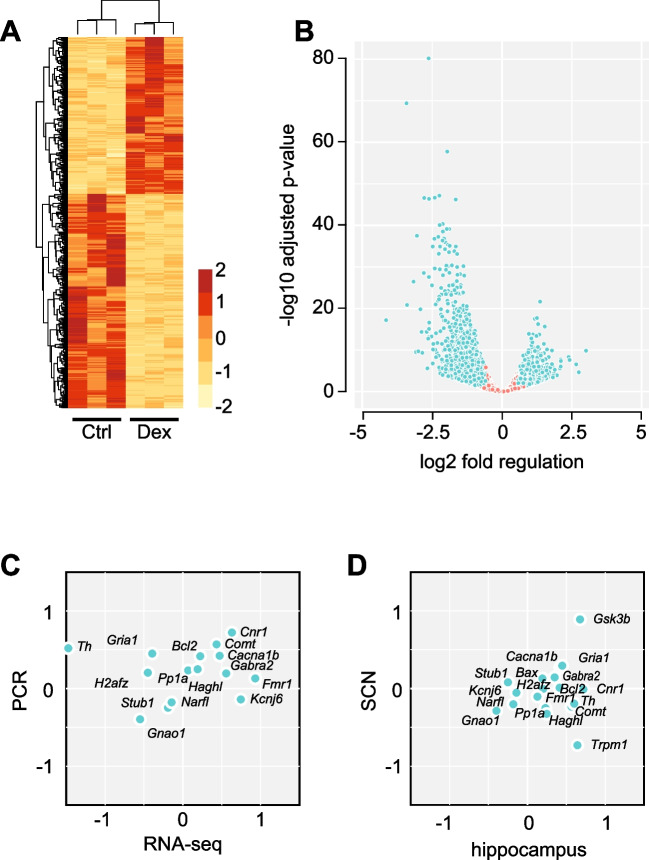
Differentially expressed genes in females exposed to Dex in utero. **A** Heatmap of differential gene expression in bulk RNA-sequencing of SCN samples. **B** Volcano plot illustrating significantly regulated mRNA species (green symbols). **C** Validation of selected DEGs by qPCR in tissue samples from SCN. **D** Validation of differential gene expression regulation between hippocampus and the SCN. Horizontal and vertical axes represent log2 fold regulation. mRNA species with similar differential regulation map in top-right and bottom-left quadrants

The delayed re-entrainment of spontaneous activity in Dex-females prompted further investigation of SCN function. We addressed the differences in gene expression in the SCN by bulk RNA-sequencing on SCN samples from female mice. Out of the 40,838 genes identified by bulk RNA-sequencing, we removed 9071 genes labelled as clock-controlled genes (CCG) (http://cgdb.biocuckoo.org/download.php) to avoid false positive discoveries due to desynchronisation of the SCN between mice (Fig. 6A). We found 2312 differentially expressed genes (DEGs) (935 upregulated) surviving FDR correction (Fig. 6B), which we further analysed by means of gene ontology (GO) term analysis using an enrichment map. Most enriched terms of the biological process were among cell morphology, neuron development and regulation of the nervous system development. Three out of the top 5 enrich GO terms were directly associated with neuron development and synapse organisation as well as a cluster involved in cell projection organisation (Supplementary Fig. S6 and S7). Next, we evaluated the impact on signalling using SPIA, and found 6 pathways significantly (after Bonferroni correction) altered in Dex-exposed females: Alzheimer’s disease, glutamatergic synapse, dopaminergic synapse, GABAergic synapse, long-term potentiation and Parkinson’s disease (Supplementary Table 4).

The decrease in dopamine signalling was particularly relevant in this context because it may explain both spontaneous hyperactivity [49] and delayed re-entrainment of activity onset [19, 20] observed in Dex-exposed females. We therefore focused further analyses on the regulation of gene expression related to dopamine signalling. We used the International Mouse Phenotype Consortium (IMPC) database and selected genes where the phenotype described in knockout models matched behavioural findings in our model. Genes selected from the IMPC database were matched with genes found in the SPIA pathways and with genes found in database ranking genes associated with ADHD based on validation in published studies. Lastly, we used the list of differentially methylated genes identified in neural stem cells exposed to Dex [44] (Table 1). We hypothesized that transcriptional changes induced by Dex exposure would result in similar differential gene expression patterns in other brain regions. To test this hypothesis, we measured the mRNA expression by means of qPCR in tissue samples from SCN and hippocampus. Differential expression of individual genes matched 73.3% between SCN qPCR and SCN bulk-sequencing (Fig. 6C) and 50% between the SCN and hippocampus qPCR (Fig. 6D; see also Supplementary Fig. S8 for mapping onto signalling pathways).

**Table 1:**
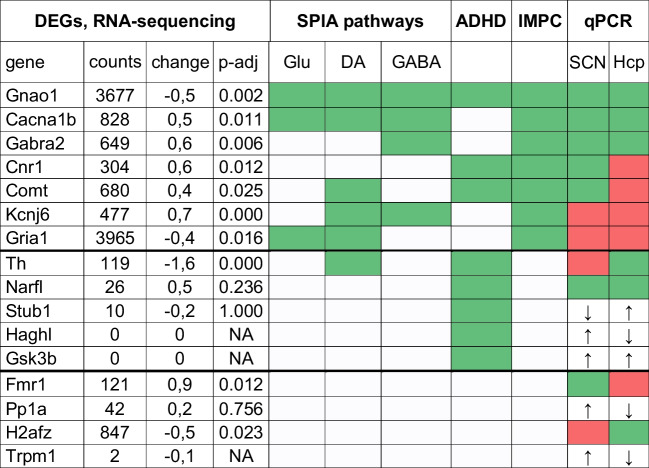
Identification of relevant genes in relation to phenotype alterations in Dex-exposed females

*ADHD,* list of genes associated with ADHD; *IMPC*, international mouse phenotype consortium; phenotypes in KO lines consistent with our findings and direction of change in bulk RNA-sequencing; qPCR validation in SCN vs. RNA-sequencing data; match – similar regulation in SCN and hippocampal formation

## Discussion

We found that exposure to excess GC by the end of the gestation period induces sex-specific long-term behavioural alterations in mice. Specifically, females exposed to Dex in utero do not develop late-onset depression-like behaviour as males do [33–35], instead they display spontaneous hyperactivity, a phenotype compatible with that observed in ADHD mouse models. Gene expression analyses suggest decreased dopamine signalling in Dex-exposed females, which may account for the overall phenotype. However, the evidence is largely circumstantial, and further experiments are required to ascertain the ADHD-like phenotype and the underlying mechanisms. In addition, variations in behavioural traits across mouse strains may confuse specific phenotypical alterations associated with human psychiatric disorders. Immobility time in FST is widely accepted as useful screening for response to antidepressants, but there are important differences between strains in baseline behaviour (reviewed in [50]), as well as in the response to antidepressants [51–53]. Therefore, the applicability of specific behavioural tests may be limited to specific mouse strains.

We implemented a robust analysis pipeline for home cage activity to capture patterns in the organisation of behaviour and investigated the sexual dimorphism in the dynamics of adaptation to a shifted light–dark cycle. Monitoring activity in the home cage in different contexts contributed distinct insights into the long-term effects of prenatal exposure to Dex. In rodents, circadian patterns of activity arise from the interaction of several components, including light–dark cycle, circadian drive by internal clock and social interactions [46]. Baseline recordings illustrate the behaviour under constant photic entrainment, and we expect minimal variations across consecutive days under the assumption that equilibrium among the mechanisms regulating circadian entrainment yields a consistent circadian pattern. In males, prenatal exposure to Dex had a significant impact on circadian entrainment [35], but rather limited effects on the organisation of home cage behaviour. In contrast, Dex females exhibit widespread changes in the organisation of home cage behaviour as compared to controls, consistent with spontaneous hyperactivity. Noteworthy, we found constitutive hyperactivity in a familiar environment (home cage, in baseline conditions)—a feature often observed in ADHD patients, but not consistently reproduced in established genetically modified animal models for ADHD [54]. The changes following the phase shift illustrate the adaptation in response to an abrupt change in the timing of transitions between light and dark. Photic re-entrainment of spontaneous activity relies on a functional SCN, and earlier reports have shown that the re-entrainment of clock gene oscillations in the SCN follows closely the re-entrainment of circadian activity in single-housed animals [55]. Recent data suggest that single housing blunts neuronal activation in the dorsal SCN and reduces spontaneous locomotor activity in male mice particularly under constant photic entrainment [56]. In our experimental setting, it appears that group-housed mice re-entrain spontaneous activity considerably faster than single-housed mice, namely within 3–4 LD cycles [33, 35] as compared to about 7 days as typically reported in the literature [19]. Given the strength of light as an entraining stimulus, one could expect a transient increase in synchronisation of activity within the cage during the first light–dark cycles (as observed in control females, but not in control males). In contrast, the synchronisation of activity within the cage drops in both males and females exposed to Dex following the phase shift. In Dex-exposed males, the decrease in synchronisation is in line with the transient loss of circadian periodicity we have described earlier [35]. In other words, the Dex-exposed males follow rather passively the light–dark cycle (as suggested by the stronger synchronisation of activity at baseline), and the weaker coupling of oscillators downstream from SCN prolongs the time to restore the synchronisation of activity among the animals in the same cage, as the adaptation to shifted LD cycle is not driven by a strong external entrainer. The decrease in social behaviour we have documented earlier [33] may also contribute to this effect. In Dex-exposed females, the delayed re-entrainment is associated with the drop in synchronisation of activity. Given the preserved coupling between SCN and downstream oscillators, this suggests an alteration further upstream interfering with the photic entrainment of the SCN. Delayed photic re-entrainment of activity onset can be accounted for by increased serotonin signalling [57], or decreased dopamine signalling [19, 58] in the SCN (reviewed in [59]). The former leads to tonic inhibition of axon terminals in the RHT and decreased postsynaptic glutamate signalling [60], but is not supported by the increase in glutamate signalling in the SCN of Dex-females as suggested by differential gene expression. Instead, SPIA analysis of differentially expressed genes points to significant inhibition of dopamine signalling in the SCN of Dex-exposed females.

We have designed a procedure for feature extraction to characterise the organisation of behaviour and used the feature space to evaluate differences at baseline as well as following the dynamic changes during re-entrainment. At baseline, we identified several significantly changed features accounting for the overall decrease in activity in males. In agreement with our previous report [33], Dex-exposed males are generally less active and display altered social behaviour as compared to control mice. Interestingly, re-entrainment in males is not accompanied by extensive changes in the organisation of behaviour, and the limited significant alterations identified are similar in Dex-exposed and controls and persisted throughout the monitoring period. However, the impact of phase shift appears blunted in Dex-exposed mice as compared to controls when all features are used as input for UMAP. This suggests that the overall organisation of behaviour in Dex-exposed males is virtually insensitive to changes in the timing of light and dark onset, and matches the photic entrainment of active phase onset, in agreement with the uncoupling downstream from SCN [35]. In contrast, Dex-exposed females travel longer distance and display more intense bouts of activity, suggesting baseline hyperactivity. The organisation of behaviour in Dex-exposed females appears more stable than in controls, and the patterns of changes do not follow the entrainment of active phase onset. This suggests that the overall control of the organisation of behaviour is preserved after the phase shift, but is independent of photic entrainment, in agreement with uncoupling upstream of SCN. A relevant technical aspect is that we have used hard boundaries between subjective day and night before running the feature extraction procedure. In other words, the time series of activity were assigned to light or dark phase using clock time (and light on or off status, implicitly) as unique criterion. Therefore, phase-specific behaviours may be assigned to the opposite phase and thereby blur the difference between light and dark (leakage of phase-specific behaviours). For instance, anticipatory behaviour such as an increase in activity ahead of dark onset is included in the light phase summary statistics and increases the total activity count for light phase. The leakage of phase-specific behaviours following the phase shift (e.g. prolonged transitions or mistimed bouts of activity) may account for deviations from baseline, as observed particularly in control females, where the organisation of behaviour in light and dark map closer after phase shift. However, the changes observed in all other groups are not consistent with the hypothesis of leakage of behaviours due to hard boundaries. Another relevant phenomenon to consider is masking (i.e. behavioural response to changes in environment, reviewed in [61]; positive masking = activation upon light off; negative masking = suppression of activity by light on). Positive and negative masking may obscure the re-entrainment and can represent strong enough events to drive re-entrainment by activation of dopaminergic signalling [61] as well as by driving episodic bursts of clock gene expression in the SCN [62]. Of note, the onset of dark phase allows nocturnal animals to become active but does not necessarily induce activity [63]. In males, particularly in Dex-exposed, masking appears to play an important role in photic re-entrainment, since the delay between activity onset and circadian peak is constant. In contrast, masking is virtually absent in female mice after advancing the onset of dark phase. In control females, re-entrainment of onset and circadian peak of activity have similar timelines (as illustrated by the constant delay between onset and circadian peak of activity) and are accompanied by larger deviations from baseline than we observed in males in both subjective day and subjective night. In Dex-exposed females, the re-entrainment of circadian peak of activity is delayed as compared to the onset of activity, suggesting a robust drive of the internal clock on spontaneous activity. This is supported by clock gene expression analysis, which indicates preserved photic entrainment, and robust coupling between SCN and peripheral oscillators, in contrast to the altered downstream coupling we observed in males [35].

We have shown earlier that transient exposure to Dex induces global DNA hypomethylation in rat embryonic cortical neural stem cells and in the cortex of mouse pups exposed to Dex in utero [44]. While our earlier investigation did not provide any indication of sex differences because the cells were isolated from pups regardless of sex, it points to widespread changes in gene expression regulation in the brain which may affect behaviour. Our decision to remove the clock-controlled genes before identifying DEGs may have restricted the number of significantly regulated genes. Oscillations in gene expression enhance between-subject variability in cross-sectional analyses and do not support reliable interpretations of significant differential regulation. In addition, one can also assume that differences in mRNA expression levels are due to differential regulation by upstream transcription factors and signalling pathways. We therefore focused on pathway analysis, such as dopaminergic signalling, rather than on specific genes. Consistent differences in gene expression between SCN and hippocampus suggest that altered dopaminergic signalling is not restricted to SCN and may be the result of altered gene expression regulation by epigenetic effects. Notably, baseline hyperactivity is consistently represented in the phenotypes associated with the genes with matching regulation within the SCN and the hippocampus. We found a consistent upregulation of *Gsk3b* expression in both SCN and hippocampus. Gsk3b destabilizes the molecular clock by promoting Bmal1 ubiquitination and subsequent degradation [64, 65], and increased Gsk3b activity has been described in several psychiatric disorders with documented impairment in circadian clock function (e.g. bipolar disorder, schizophrenia, ADHD, and depression) [66]. The activity of Gsk3b is tightly regulated by phosphorylation, and decreased dopaminergic signalling leads to inactivation [67]. Dopamine signalling via D2 receptors inactivates Akt and thereby reduces inhibitory phosphorylation of Gsk3b, while active Gsk3b makes the molecular clock more flexible by weakening the coupling between oscillations in clock gene expression [67]. This may explain why dopaminergic projections from VTA to the SCN facilitate the photic re-entrainment of activity [19], and can account for delayed re-entrainment of activity onset in Dex-exposed females. Therefore, while constitutive upregulation of Gsk3b may be relevant in relation to the global phenotype, its impact on circadian entrainment may be mitigated by the inactivation due to decreased dopamine signalling. Altered dopaminergic signalling results in strong sexually dimorphic outcomes, which potentially contribute to sex-related differences in prevalence of psychiatric disorders [68]. In Dex-exposed females, the decreased dopaminergic signalling may explain both the delayed re-entrainment [19, 58], and spontaneous hyperactivity [49]. This is in line with the circadian alterations described in ADHD patients, where bright light therapy can restore and maintain the circadian rhythm close to normal (reviewed in [69]).

Taken together, our data suggests that photic entrainment of activity is to a large extent independent from the control of the organisation of behaviour in both males and females exposed to Dex in utero. The core difference is that photic entrainment of activity is weakened in males by uncoupling of downstream oscillators [35], and in females by weaker photic entrainment due to decreased dopamine signalling in the SCN. Our study also highlights sexual dimorphism in susceptibility to neurodevelopmental insults and the onset of multifactorial psychiatric disorders.

### Supplementary Information

Below is the link to the electronic supplementary material.

Supplementary file1 (DOCX 1040 KB)

Supplementary file2 (PDF 201 KB)

Supplementary file3 (PDF 449 KB)

## Data Availability

The datasets generated or analysed in this experiment are not publicly available but are available from the corresponding author upon reasonable request. The software code, developed in Matlab™ and R, can be made available upon reasonable request.
