# Supplementary material for: Sex Differences in Long-term Outcome of Prenatal Exposure to Excess Glucocorticoids—Implications for Development of Psychiatric Disorders
Source: Mol Neurobiol. 2023 Aug 10;60(12):7346–61. doi: 10.1007/s12035-023-03522-5 (PMC10657788; doi:10.1007/s12035-023-03522-5)
Supplement: Supplementary file 1 — Supplementary file1 (DOCX 1040 KB) [file 12035_2023_3522_MOESM1_ESM.docx]

**Sex-differences in long-term outcome of prenatal exposure to excess glucocorticoids – implications for development of psychiatric disorders**

Frederik Elberling^1*^, Stefan Spulber^1*CA^, Raj Bose^1^, Hoi Yee Keung^2^, Virpi Ahola^2^, Zongli Zheng^2^, Sandra Ceccatelli^1^

^1^ - Department of Neuroscience, Karolinska Institutet, Stockholm, Sweden

^2^ - Ming Wai Lau Centre for Reparative Medicine, Karolinska Institutet, Hong Kong Special Administrative Region of People’s Republic of China

**Supplementary Material Contents:**

**Supplementary Fig. S1** Data-driven definition of threshold between active and inactive (rest intervals) visits.

**Supplementary Fig. S2** Total activity count over 24h (complete LD cycle) at baseline.

**Supplementary Fig. S3** Illustration of clusters identified using affinity propagation (AP) clustering based on the pattern of variation after an abrupt advance in dark onset by 6h.

**Supplementary Fig. S4** Normalized individual features in the UMAP representation.

**Supplementary Fig. S5** Calculation of distances between individual points based on the UMAP mapping of organization of behaviour.

**Supplementary Fig. S6** Gene ontology (GO) term analysis for differentially expressed genes (external file)

**Supplementary Fig. S7**Top GO terms (external file).

**Supplementary Fig. S8** Overlap among differentially expressed genes included in significantly changed signalling pathways identified by SPIA.

**Supplementary Table 1** List of features used for AP-clustering and UMAP.

**Supplementary Table 2** Primer sequences used for rt-qPCR on mRNA isolated from brain tissue samples.

**Supplementary Table 3** R-studio packages used.

**Supplementary Table 4** Significantly altered signalling pathways identified by SPIA pathway analysis.

**Supplementary Information**


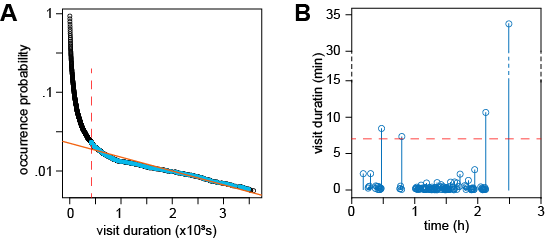


**Supplementary Fig. S1.** (A) Detection of threshold between active (short, assumed to be part of activity bouts) and inactive (long, associated with resting and sleeping) visits. The dashed vertical line indicates the threshold (~7 min duration) identified as the point where the log-normal plot start deviating significantly from exponential fit. (B) Illustration of a sequence of 274 datapoints spanning ~3 h. Note the alternations of clusters of short visits forming activity bouts, and very long inactive intervals. The sequence of consecutive short visits between two resting intervals (visits longer than the threshold) is defined as “trip”. The horizontal dashed line corresponds to the threshold in (A).


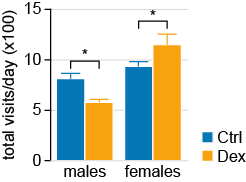


**Supplementary Fig. S2.** Activity levels over 24-h LD cycle.

**Supplementary Fig. S3 Affinity propagation clustering**


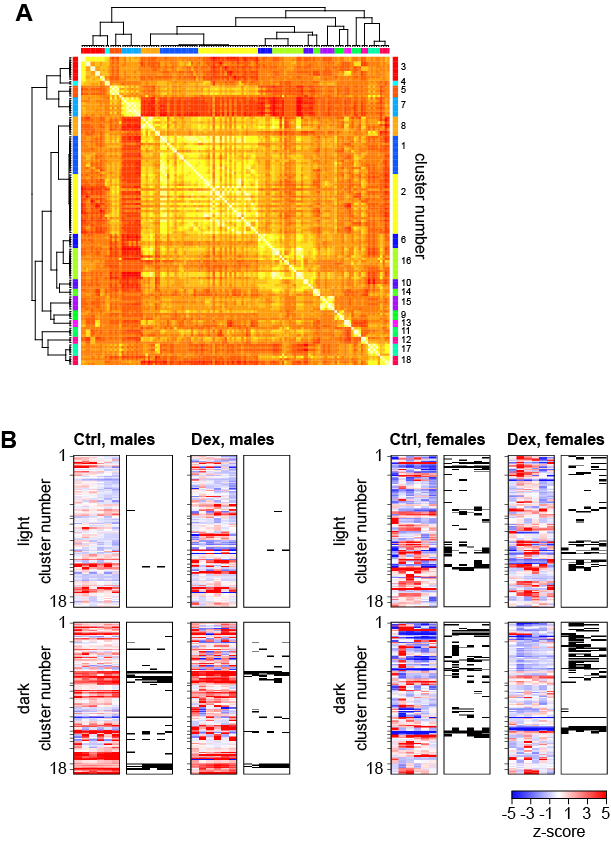


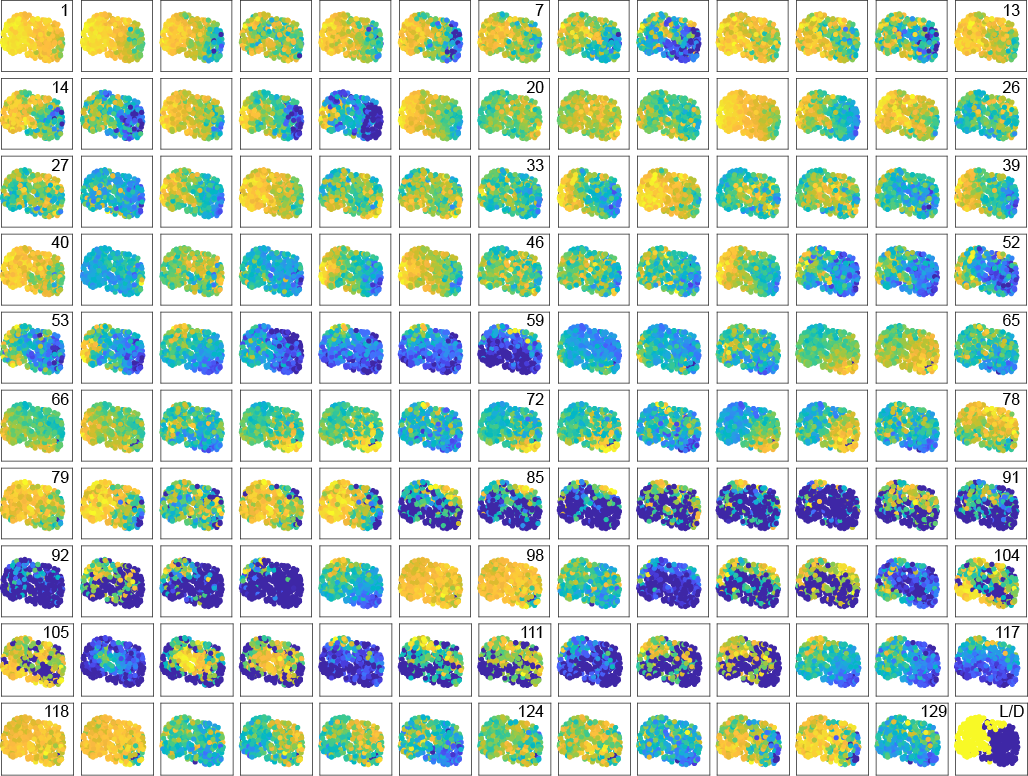
**Supplementary Fig. S4.** Normalized individual features in the UMAP representation. The numeric identifiers correspond to feature numbers in Supplementary Table 1. The bottom right map depicts the separation between light and dark behaviours (yellow and blue, respectively). Note there are several features displaying gradients across dark and light behaviours (*e.g*., 2, 3, 6, 8, 9, 17, 18, 75, 76, 121).


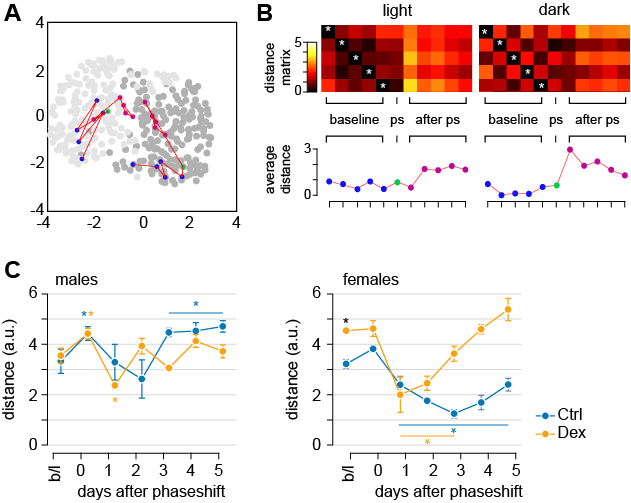
**Supplementary Fig. S5.** Calculation of trajectories in UMAP plane for one individual mouse (control female). (A) Darker dots indicate behaviour recorded during dark phase. Individual light and dark phases are recorded in the order indicated by the line. (B) Illustration of distance matrix used for estimation of deviation from baseline profile (top). Since the algorithm used to construct the UMAP treats individual datapoints as independent (does not take into account the sequence of days recorded), we assigned equal weights to baseline datapoints and calculated average Euclidean distances from baseline for each point (bottom). * - distances not used in calculating averages (distance between identical points is equal to 0). (C) Similar calculations were used for estimating the distance between consecutive light and dark behaviour for each mouse. Note the average distance between light and dark behaviour at baseline is about 3 times higher than the average distance between light or dark, respectively (see Fig. 4B in the main text and (B) for reference). Dex-males display significant biphasic variations before returning to baseline level, while controls display an increase in distance between light and dark after the phase shift. In contrast, Dex-females display a transient decrease following the phase shift, while in control females the difference decreases to levels comparable to the difference between light or dark behaviours, before slowly returning to baseline levels.

**Supplementary Fig. S6** Gene ontology (GO) term analysis for differentially expressed genes. Explore separate file for details.

**Supplementary Fig. S7** Top GO terms. Explore separate file for details


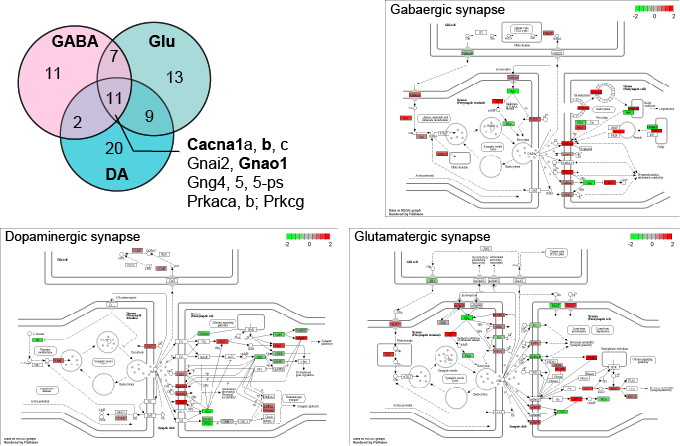


**Supplementary Fig. S8** Overlapping between SPIA signalling pathways. Genes in bold typeface are included in the main text, Table 1

^30^

**Supplementary Table 1**

Each feature is measured over 12h of subjective night or subjective day.

|  | Variables | Activity | Description | Cluster # |
| --- | --- | --- | --- | --- |
| 1 | Trip_C0 | Trip | Time spent alone | 1 |
| 2 | Trip_C1 | Trip | Time spent with another animal | 1 |
| 3 | Trip_C_All | Trip | Time spent with more than one animal | 1 |
| 4 | Trip_Nest_0 | Trip | Time spent alone in Nest | 1 |
| 5 | Trip_Nest_1 | Trip | Time spent with another animal in Nest | 1 |
| 6 | Trip_Nest_All | Trip | Time spent with more than one animal in Nest | 2 |
| 7 | Trip_Adjacent_0 | Trip |  | 2 |
| 8 | Trip_Adjacent_1 | Trip |  | 1 |
| 9 | Trip_Adjacent_All | Trip |  | 1 |
| 10 | Trip_Diagonal_0 | Trip |  | 1 |
| 11 | Trip_Diagonal_1 | Trip |  | 1 |
| 12 | Trip_Diagonal_All | Trip |  | 1 |
| 13 | Trip_Across_0 | Trip |  | 1 |
| 14 | Trip_Across_1 | Trip |  | 1 |
| 15 | Trip_Across_All | Trip |  | 9 |
| 16 | Trip_Middle_0 | Trip |  | 2 |
| 17 | Trip_Middle_1 | Trip |  | 2 |
| 18 | Trip_Middle_All | Trip |  | 2 |
| 19 | Trip_Total_Antenna | Trip | Number of antennas | 2 |
| 20 | Trip_Time_Avg | Trip | Average time spent per antenna | 3 |
| 21 | Trip_Time_SD | Trip | SD of time spent | 3 |
| 22 | Trip_Time_Skew | Trip | Skewness of time spent | 2 |
| 23 | Trip_Total_Distance | Trip | Total distance covered | 2 |
| 24 | Trip_Nest | Trip | Number of visits in Nest | 2 |
| 25 | Trip_Nest_Time | Trip | Time spent in Nest | 1 |
| 26 | Trip_Nest_Avg | Trip | Average time spent per antenna in Nest | 3 |
| 27 | Trip_Nest_SD | Trip | SD of time spent in Nest | 3 |
| 28 | Trip_Nest_Skew | Trip | Skewness of time spent in Nest | 2 |
| 29 | Trip_Adjacent | Trip |  | 2 |
| 30 | Trip_Adjacent_Time | Trip |  | 1 |
| 31 | Trip_Adjacent_Avg | Trip |  | 3 |
| 32 | Trip_Adjacent_SD | Trip |  | 3 |
| 33 | Trip_Adjacent_Skew | Trip |  | 2 |
| 34 | Trip_Diagonal | Trip |  | 2 |
| 35 | Trip_Diagonal_Time | Trip |  | 1 |
| 36 | Trip_Diagonal_Avg | Trip |  | 3 |
| 37 | Trip_Diagonal_SD | Trip |  | 3 |
| 38 | Trip_Diagonal_Skew | Trip |  | 2 |
| 39 | Trip_Across | Trip |  | 2 |
| 40 | Trip_Across_Time | Trip |  | 1 |
| 41 | Trip_Across_Avg | Trip |  | 3 |
| 42 | Trip_Across_SD | Trip |  | 3 |
| 43 | Trip_Across_Skew | Trip |  | 2 |
| 44 | Trip_Middle | Trip |  | 2 |
| 45 | Trip_Middle_Time | Trip |  | 2 |
| 46 | Trip_Middle_Avg | Trip |  | 4 |
| 47 | Trip_Middle_SD | Trip |  | 4 |
| 48 | Trip_Middle_Skew | Trip |  | 2 |
| 49 | Trip_Total_Return | Trip | Number of roundtrips (return to same antenna) | 2 |
| 50 | Trip_Nest_Return | Trip | Roundtrips originating in nest | 2 |
| 51 | Trip_Adjacent_Return | Trip |  | 2 |
| 52 | Trip_Diagonal_Return | Trip |  | 2 |
| 53 | Trip_Across_Return | Trip |  | 2 |
| 54 | Trip_Middle_Return | Trip |  | 2 |
| 55 | Number_Trips | Trip | Total number of trips | 16 |
| 56 | NN | Trip | number of nest to nest trips | 8 |
| 57 | NR | Trip | number of nest to rest trips | 16 |
| 58 | RN | Trip | number of rest to nest trips | 16 |
| 59 | RR | Trip | number of rest to rest trips | 16 |
| 60 | T_Avg_Antenna | Trip | average number of antennas per trip | 5 |
| 61 | T_SD_Antenna | Trip | SD in number of antennas per trip | 5 |
| 62 | T_Skew_Antenna | Trip | skewness number of antennas per trip | 6 |
| 63 | T_Avg_Time | Trip | average time per trip | 7 |
| 64 | T_SD_Time | Trip | SD time per trip | 7 |
| 65 | T_Skew_Time | Trip | Skewness time per trip | 6 |
| 66 | T_Avg_Distance | Trip | average distance per trip | 5 |
| 67 | T_SD_Distance | Trip | SD distance per trip | 5 |
| 68 | T_Skew_Distance | Trip | Skewness per trip | 6 |
| 69 | T_Avg_Alone | Trip | average time alone during trips | 7 |
| 70 | T_SD_Alone | Trip | SD time alone during trips | 7 |
| 71 | T_Skew_Alone | Trip | skewness of time alone during trips | 6 |
| 72 | T_Avg_p1 | Trip | with another animal | 7 |
| 73 | T_SD_p1 | Trip |  | 7 |
| 74 | T_Skew_p1 | Trip |  | 6 |
| 75 | T_Avg_All | Trip | with more than one animal | 7 |
| 76 | T_SD_All | Trip |  | 7 |
| 77 | T_Skew_All | Trip |  | 6 |
| 78 | Rest_C0 | Rest | Time alone | 18 |
| 79 | Rest_C1 | Rest | Time with one animal | 16 |
| 80 | Rest_C_All | Rest | with more than one animal | 8 |
| 81 | Rest_Nest_0 | Rest | Time spent alone in Nest | 18 |
| 82 | Rest_Nest_1 | Rest | Time spent with another animal in Nest | 8 |
| 83 | Rest_Nest_All | Rest | Time spent with more than one animal in Nest | 8 |
| 84 | Rest_Adjacent_0 | Rest |  | 16 |
| 85 | Rest_Adjacent_1 | Rest |  | 16 |
| 86 | Rest_Adjacent_All | Rest |  | 11 |
| 87 | Rest_Diagonal_0 | Rest |  | 16 |
| 88 | Rest_Diagonal_1 | Rest |  | 16 |
| 89 | Rest_Diagonal_All | Rest |  | 16 |
| 90 | Rest_Across_0 | Rest |  | 9 |
| 91 | Rest_Across_1 | Rest |  | 9 |
| 92 | Rest_Across_All | Rest |  | 9 |
| 93 | Rest_Middle_0 | Rest |  | 15 |
| 94 | Rest_Middle_1 | Rest |  | 15 |
| 95 | Rest_Middle_All | Rest |  | 15 |
| 96 | Rest_Total_Antenna | Rest | Number of antennas | 16 |
| 97 | Rest_Time_Avg | Rest | Average time spent per antenna | 17 |
| 98 | Rest_Time_SD | Rest | SD of time spent | 17 |
| 99 | Rest_Time_Skew | Rest | Skewness of time spent | 10 |
| 100 | Rest_Nest | Rest | Number of visits in Nest | 11 |
| 101 | Rest_Nest_Time | Rest | Time spent in Nest | 11 |
| 102 | Rest_Nest_Avg | Rest | Average time spent per antenna in Nest | 11 |
| 103 | Rest_Adjacent | Rest |  | 12 |
| 104 | Rest_Adjacent_Time | Rest |  | 12 |
| 105 | Rest_Adjacent_Avg | Rest |  | 12 |
| 106 | Rest_Diagonal | Rest |  | 13 |
| 107 | Rest_Diagonal_Time | Rest |  | 13 |
| 108 | Rest_Diagonal_Avg | Rest |  | 13 |
| 109 | Rest_Across | Rest |  | 14 |
| 110 | Rest_Across_Time | Rest |  | 14 |
| 111 | Rest_Across_Avg | Rest |  | 14 |
| 112 | Rest_Middle | Rest |  | 15 |
| 113 | Rest_Middle_Time | Rest |  | 15 |
| 114 | Rest_Middle_Avg | Rest |  | 15 |
| 115 | Rest_Number_Rest | Rest |  | 16 |
| 116 | Rest_Nest | Rest | number of resting sessions in Nest | 8 |
| 117 | Rest_Rest | Rest | number of resting sessions outside the Nest | 16 |
| 118 | R_Avg_Time | Rest | average duration of resting bouts | 17 |
| 119 | R_SD_Time | Rest | SD time | 17 |
| 120 | R_Skew_Time | Rest | Skewness time | 10 |
| 121 | R_Avg_Alone | Rest | Average time Alone | 18 |
| 122 | R_SD_Alone | Rest | SD time alone | 18 |
| 123 | R_Skew_Alone | Rest | Skewness time alone | 8 |
| 124 | R_Avg_p1 | Rest | with another animal | 5 |
| 125 | R_SD_p1 | Rest |  | 17 |
| 126 | R_Skew_p1 | Rest |  | 10 |
| 127 | R_Avg_All | Rest | with more than one animal | 8 |
| 128 | R_SD_All | Rest |  | 8 |
| 129 | R_Skew_All | Rest |  | 10 |

**Supplementary Table 2 Primer sequences**

| gene ID | primer sequence |
| --- | --- |
| Gapdh_L | CAAGGCCGAGAATGGGAAG |
| Gapdh_R | GGCCTCACCCCATTTGATGT |
| Bmal1_L | AACCTTCCCGCAGCTAACAG |
| Bmal1_R | AGTCCTCTTTGGGCCACCTT |
| Per1_L | CCAGATTGGTGGAGGTTACTGAG |
| Per1_R | GCGAGAGTCTTCTTGGAGCAGTA |
| Cacna1b_L | CATGACGTCTTGCTTCCTGA |
| Cacna1b_R | TACAGCCACTGCTTGACAGG |
| Cnr1_L | CTGGTTCTGATCCTGGTGGT |
| Cnr1_R | TGTCTCAGGTCCTTGCTCCT |
| Comt_L | GTCATCCTGATGGCCTCACT |
| Comt_R | TACCGTCTGAGTCGTTGCTG |
| Fmr1_L | TGCCATTTCATGTCCTGTGT |
| Fmr1_R | CTTCCCTGAACTCTGCATCC |
| Gabra2_L | CAGCTGAAGCCAAGAAAACC |
| Gabra2_R | CCAAAGCCCAAAAGTAACCA |
| Gnao1_L | TCGTGATTTTCTCCCCTTTG |
| Gnao1_R | TTTTTCTCAATCGCCTTGCT |
| Gria1_L | CTAGGCTGCCTGAACCTTTG |
| Gria1_R | GGGAAGATTGAATGGAAGCA |
| Gsk3b_L | TGGCGTGTGATGTCAGGTAT |
| Gsk3b_R | TAAGCTGGCATCTGCAACAC |
| Haghl_L | AGTTGAAGCTGGGACAATGG |
| Haghl_R | CTGGGAGCACAAAGGAAGAG |
| Kcnj6_L | CAACCTCAACGGGTTTGTCT |
| Kcnj6_R | ATCCTACCATGAAGGCGTTG |
| Narfl_L | GGAGGCCTTGAGTCTGTGAG |
| Narfl_R | CTGGAGCACAGAGTCCAACA |
| Stub1_L | CGCTGCAACAGAGTCAAGAG |
| Stub1_R | CCTATGACCGCAAGGACATT |
| Th_L | CAGCTGGAGGATGTGTCTCA |
| Th_R | GGCATGACGGATGTACTGTG |

**Supplementary Table 3** Rstudio packages used

| input data | packages |
| --- | --- |
| activity recording | apcluster; cluster; data.table; factoextra; moments; R.matlab; tidyverse; umap; zoo |
| RNA-sequencing | AnnotationHub; biomaRt; clusterProfiler; DESeq2; enrichplot; ensembledb; ggrepel; org.Mm.eg.db; pathview; pheatmap; RColoBrewer; readxl; SPIA; tidyverse |

**Supplementary Table 4** SPIA pathways

| Name | DEGs | FDR-adjusted p value | Status |
| --- | --- | --- | --- |
| Alzheimer's disease | 50 | 4.25E-07 | Activated |
| Glutamatergic synapse | 40 | 1.22E-04 | Activated |
| Dopaminergic synapse | 42 | 3.08E-04 | Inhibited |
| GABAergic synapse | 31 | 4.22E-04 | Activated |
| Long-term potentiation | 27 | 5.43E-04 | Inhibited |
| Parkinson's disease | 29 | 1.43E-03 | Activated |
