# Supplementary figures and images for: Sex Differences in Long-term Outcome of Prenatal Exposure to Excess Glucocorticoids—Implications for Development of Psychiatric Disorders
Source: Mol Neurobiol. 2023 Aug 10;60(12):7346–61. doi: 10.1007/s12035-023-03522-5 (PMC10657788; doi:10.1007/s12035-023-03522-5)

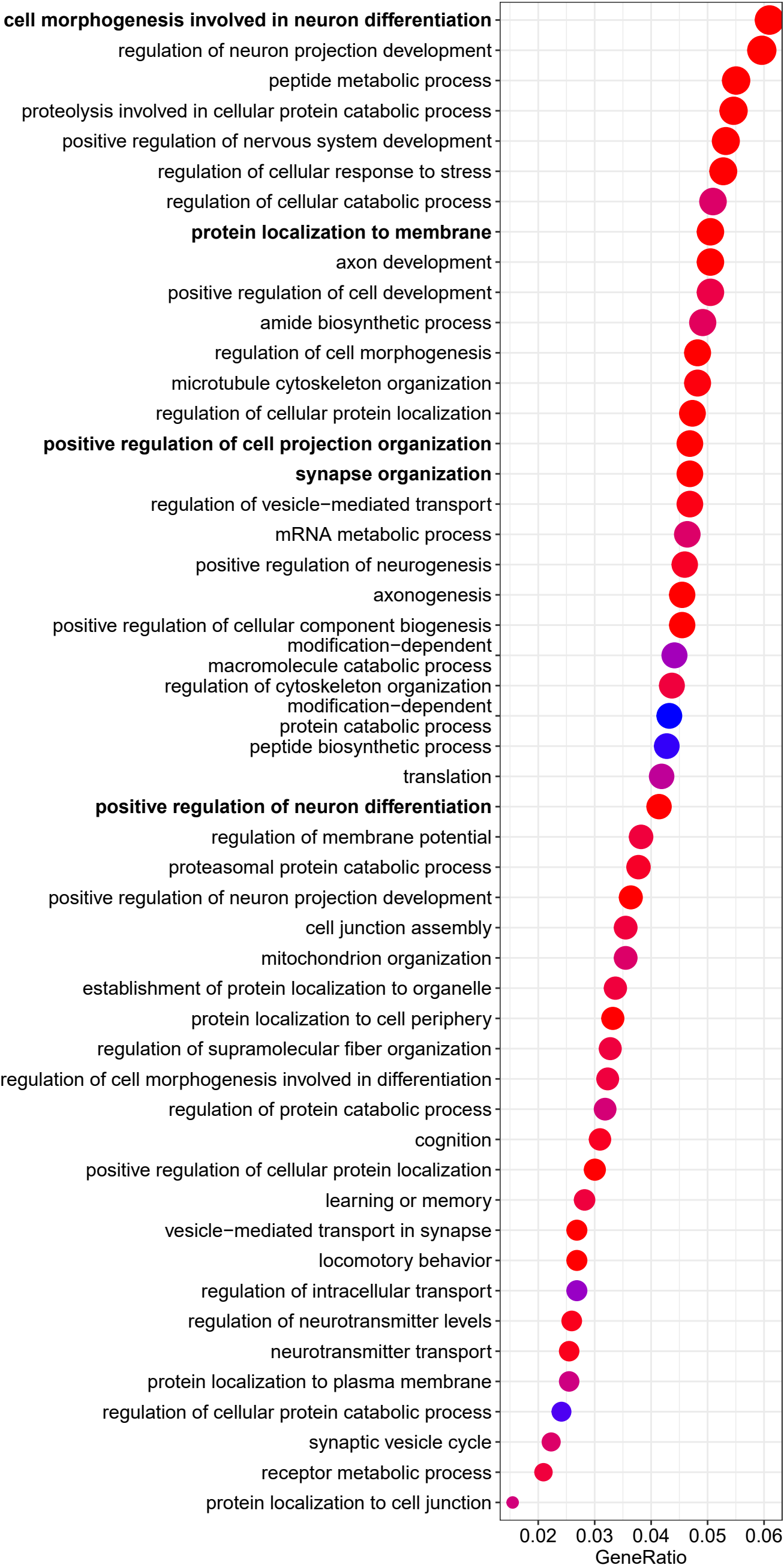

Supplement: Supplementary file 2 — Supplementary file2 (PDF 201 KB) [file 12035_2023_3522_MOESM2_ESM.pdf]

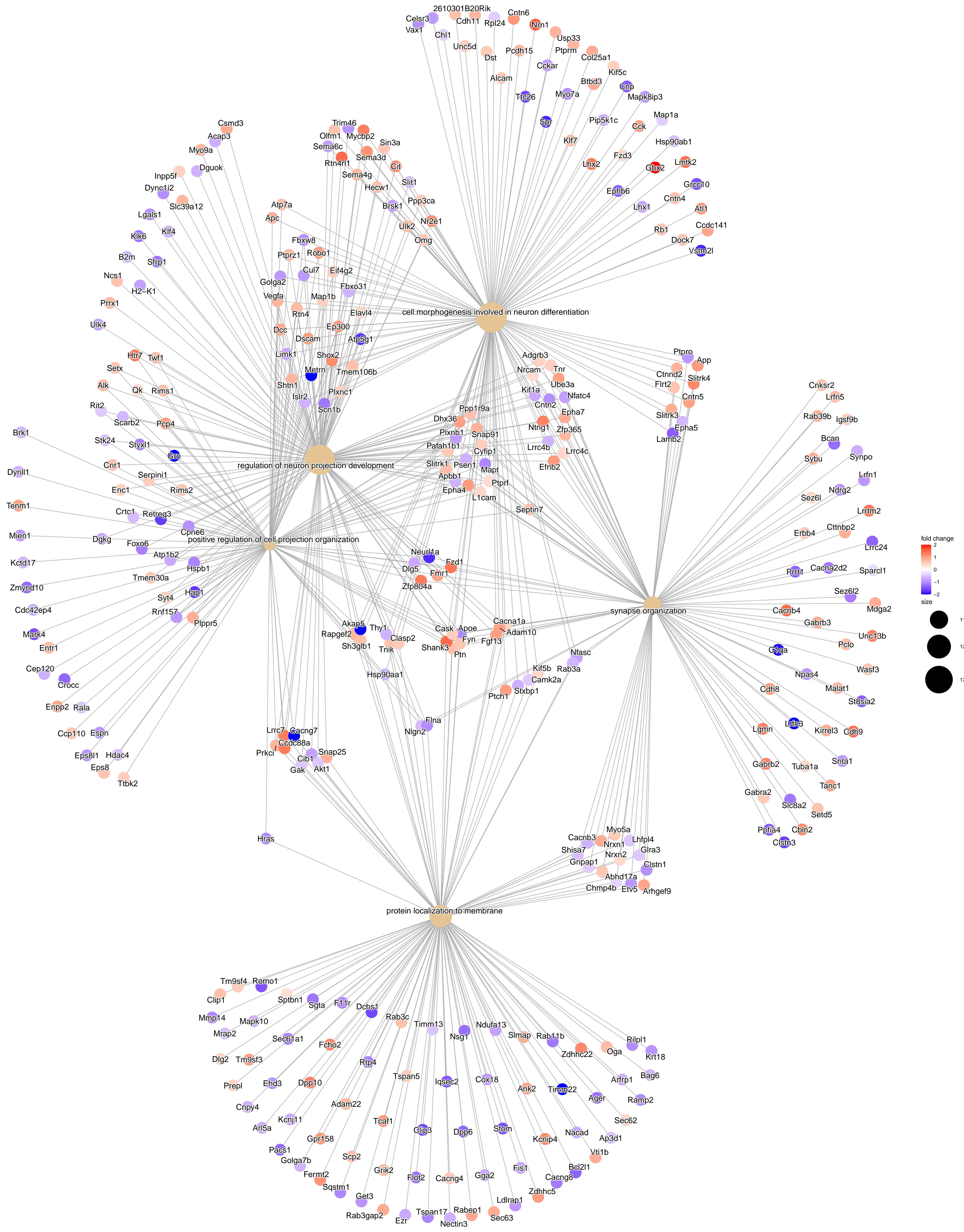

Supplement: Supplementary file 3 — Supplementary file3 (PDF 449 KB) [file 12035_2023_3522_MOESM3_ESM.pdf]
